# Uniform Fast-Kinetic Anode/Cathode Electrolyte Interphases Enable High Performance 3C Li-Metal Batteries with > 99.9% Coulombic Efficiencies

**DOI:** 10.1007/s40820-026-02088-w

**Published:** 2026-02-09

**Authors:** Qingyang Cao, Danchen Fu, Xuedong He, Yaohua Huang, Ningning Yao, Chunyu Song, Huawei Song, Chengxin Wang

**Affiliations:** State Key Laboratory of Optoelectronic Materials and Technologies, School of Materials Science and Engineering, Sun Yat-Sen (Zhongshan) University, Guangzhou, 510275 People’s Republic of China

**Keywords:** Fast charging, Electrolyte additive, Interphase regulation, Li-metal, LiFePO_4_

## Abstract

Lithium metal batteries (LMBs) represent one of the most promising energy storage systems due to unparalleled energy density. However, in commercial electrolytes, their practical high-power performance is still hampered by unstable electrolyte interfaces, leading to severe anode dendrite growth and cathode degradation. Here, 4-fluoro-3-nitrophenylboronic acid is introduced as a dual-function additive, contributing to uniform N-/F-rich interphase layers at both electrodes of the LMBs. Therefore, in the optimized electrolyte, Li-metal electrodes demonstrate enhanced plating/stripping reversibility of > 700 h (vs. 250 h at 1 mA cm^−2^ and 0.5 mAh cm^−2^) and coulombic efficiency of 98.2% (vs. 84.2%). Moreover, the corresponding LMBs achieve 99.9% capacity retention (vs. 44.7%) after 500 cycles at 3C rate, simultaneously maintaining > 99.9% coulombic efficiencies. The impressive fast-charging performance attributes to not only the uniform and compact Li deposition at the anode, but also the inhibited uncontrolled electrolyte decomposition and active species loss at the cathode due to the robust electrolyte interphases. This work highlights that proper electrolyte additive is crucial for fast-charging metal batteries.

**Supplementary Information:**

The online version contains supplementary material available at 10.1007/s40820-026-02088-w.

## Highlights

- 4-Fluoro-3-nitrophenylboronic acid, as an additive, has contributed to uniform N-/F-rich interphase layers at both electrodes of the lithium metal batteries.
- Uniform interphase layers inhibited Li dendrite growth at Li-metal anode, and alleviated uncontrolled electrolyte decomposition and active species loss at the LiFePO_4_ (LFP) cathode.
- Li ||Li cells demonstrate enhanced plating/stripping reversibility of >700 h at 1 mA cm^−2^ and 0.5 mAh cm^−2^, while Li ||LFP cells can be stably cycled for over 500 cycles at 3C rate with a capacity retention of 99.9%, simultaneously maintaining >99.9% coulombic efficiencies.

**Supplementary Information:**

The online version contains supplementary material available at 10.1007/s40820-026-02088-w.

## Graphical abstract

**Figure:**
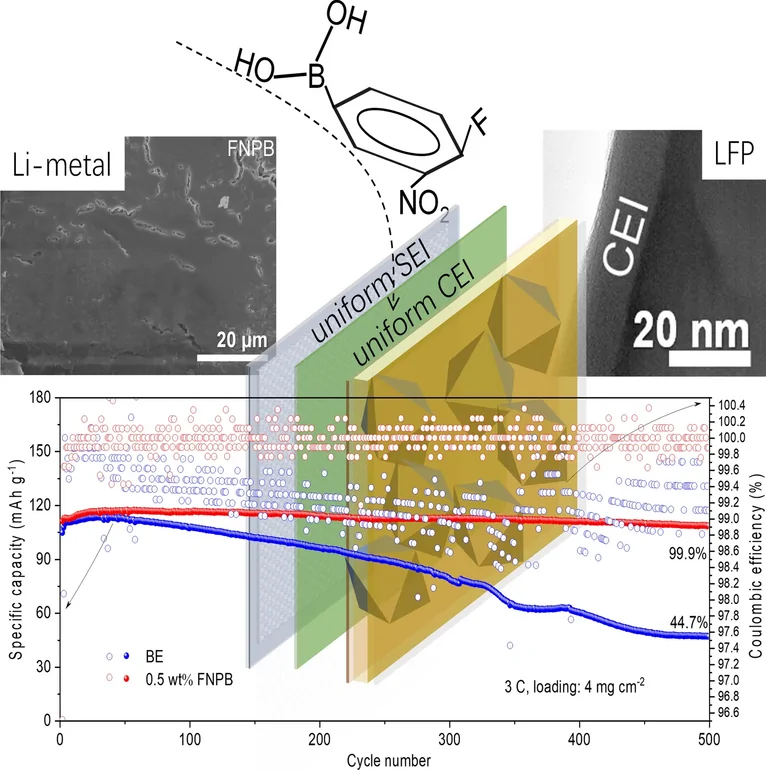

## Introduction

Lithium ion batteries have been the preferred power sources for portable electronic devices, electric vehicles, and grid-scale energy storage systems [1]. However, the energy density is limited due to single charge intercalation mechanism of the graphite anode [2]. Lithium metal, possessing ultrahigh capacity (3860 mAh g^−1^) and the lowest redox potential (− 3.04 V), is recognized as the most promising anode for high-energy–density batteries [3–6]. But the practical application of Lithium metal batteries (LMBs) faces significant challenge of inhomogeneous Li deposition which readily forms protruding dendrites. The concentrated electric field at the dendrite, known as sharp-edge effect, inevitably accelerates uneven Li deposition and electrolyte decomposition [7]. The dendrites also possibly penetrate separators, raising safety concerns of internal short circuit and thermal runaway [8]. Therefore, the issue of lithium dendrite growth should be properly addressed to achieve practical LMBs [9, 10].

Various strategies, e.g., optimizing electrolyte formulations, developing solid-state electrolytes, engineering artificial solid electrolyte interphase (SEI) layers, designing three-dimensional metal deposition frameworks, etc., have been extensively explored to control metal deposition behavior and suppress dendrite growth [8, 11–14]. Among them, a stable SEI with a variety of inorganic and organic species at the electrode–electrolyte interface is effective to inhibit metal dendrites [8, 15–23]. Some inorganic interphases in the SEIs inhibit lithium dendrite proliferation through electron tunneling prevention, uniform Li^+^ transport facilitation, and enhanced mechanical stability [24]. Specifically, the lithium fluoride (LiF) interphase usually enhances the interface stability, but its low ionic conductivity leads to sluggish interfacial kinetics and uneven lithium deposition, compromising the fast-charging capability of LMBs [25]. In contrast, the lithium nitride (Li_3_N) interphase effectively compensates for the shortcomings of LiF due to high ionic conductivity. Therefore, designing SEIs with uniform LiF and Li_3_N interphases is beneficial for achieving high-rate charging stability in LMBs [26, 27]. However, the SEIs naturally evolved in conventional electrolytes typically exhibit non-uniform thickness and composition, exacerbating localized dendrite proliferation and reducing battery cycle life [28–30].

The olivine-structured lithium iron phosphate (LiFePO_4_, denote as LFP afterward), benefiting from large theoretical capacity (~ 170 mAh g^−1^), high open-circuit voltage (3.45 V vs. Li^+^/Li), as well as remarkable structural and chemical stability, has been widely utilized as cathodes for LMBs [1, 31]. However, it faces inherently poor electron conductivity and sluggish ion diffusion process in practical application. Although the capacity of LFP can be improved by reducing the particle size and increasing the surface area, excessively exposed particles also lead to severe interface parasitic reactions, such as loss of active species by iron dissolution and structural deterioration [32, 33]. Some functional additives, e.g., vinyl sulfonyl fluoride (VSF), fluoroethylene carbonate (FEC), vinyl ethylene carbonate (VEC), lithium difluoro(oxalato)borate (LiDFOB), and vinylene carbonate (VC), and N-fluorobenzenesulfonimide (NFSI) help to form stable cathode electrolyte interphase (CEI) layers by preferential oxidation to avoid direct cathode–electrolyte contact and suppress these interface reactions [33–39]. However, conventional organic-rich CEIs cannot accommodate substantial volume changes, and exhibit insufficient anti-oxidization stability at high voltages, leading to continuous fracture during cycling [40]. Meanwhile, the additives simultaneously engineering stable SEIs and CEIs remain elusive in practical carbonate-based electrolytes.

**Scheme 1:**
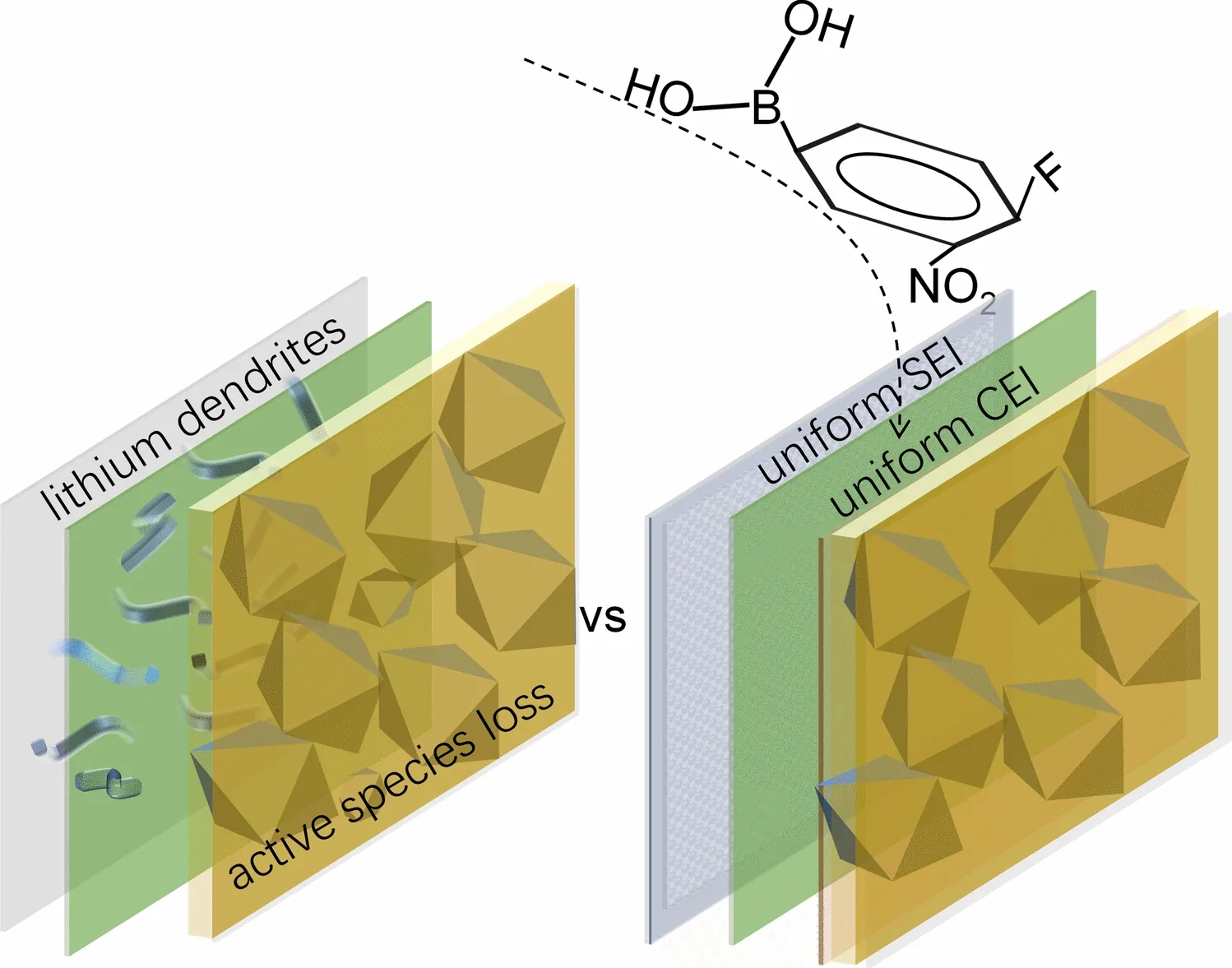
Illustration of uniform anode/cathode electrolyte interphases induced by a dual-function electrolyte additive versus the control one accompanied by anode lithium dendrites and cathode active species loss

In this study, take commercial 1 M LiPF_6_ EC/DEC (v/v = 1) electrolyte (denote as BE afterward) as an example, 4-fluoro-3-nitrophenylboronic acid (denote as FNPB afterward) is demonstrated as a novel dual-function electrolyte additive, efficiently evolving uniform electrolyte interphases at both lithium metal anode and LFP cathode. Different from F-rich or N-rich interphases evolved from additives of single functional group (such as VSF, FEC, and NFSI), FNPB featuring -BO_2_-, -NO_2_, and -F functional groups, readily forms LiF/Li_3_N/LiBO_x_ inorganic interphases with high ionic conductivity and stability, facilitating Li^+^ migration and deposition, and enhancing the cathode oxidization stability [41–43]. The synergistical effect of multiple interphases endows both SEI and CEI with enhanced stability and fast Li^+^ conductivity. As illustrated in Scheme 1, FNPB undergoes controllable decomposition at interfaces, generating uniform, stable SEI and CEI layers that reduce electrolyte consumption and enhance lithium-ion transport kinetics, in sharp contrast to anode lithium dendrites and cathode active species loss in common commercial electrolytes. Consequently, both Li-metal symmetric cells and the devices substantiate significantly enhanced reversibility and coulombic efficiencies, especially at fast-charging rates.

## Experimental Section

### Preparation of Electrolytes and LFP Cathodes

The 4-fluoro-3-nitrophenylboronic acid (FNPB, 95%) was purchased from Shanghai Macklin Biochemical Technology Co., Ltd. The BE electrolyte was purchased from DoDo Chemistry. The electrolyte was prepared by adding proper amount FNPB into the BE electrolyte, in which the mass percent of additive is controlled at 0, 0.25, 0.5, and 1 wt%. Lithium electrodes (400 μm thickness, 14 μm diameter) were purchased from China Energy Lithium Co., Ltd. The LFP cathodes were prepared by following steps. The LFP powder (Canrd), acetylene black and polyvinylidene difluoride were mixed in a weight ratio of 8:1:1, then added in N-methyl-2-pyrrolidone with stirring for 12 h. The electrodes were fabricated by pasting the slurry mixture on aluminum foil by an automatic thick film coater (AFA-I). The coating film was dried in a vacuum chamber at 80 ℃ for 12 h. Subsequently, the foil was compressed using an electromotive roller (MR-100A) and sectioned to specified dimensions (14 μm diameters) with a coin-type cell microtome (T-06). The average active material loading is around 4 mg cm^−2^. All materials were used as received.

### Physiochemical Characterization

The corresponding morphology, composition, and chemical bonding were characterized by scanning electron microscope (SEM, HITACHI, 30 kV), transmission electron microscope (TEM, FEI Tecnai G2 F30, 300 kV), energy dispersion X-ray spectrometer (EDS), and X-ray photoelectron spectrometer (XPS, Nexsa), respectively. Time-of-flight secondary ion mass spectroscopy (TOF–SIMS) depth profiling was also performed to get insights of elements involved in the surface components of Li-metal and LFP after 50 or 100 cycles. Raman spectra of the electrolytes were obtained using a RENISHAW inVia Qontor with a 633 nm laser source.

### Electrochemical Characterization

Li symmetric cells were assembled with Celgard 2500 and 30 μL electrolyte in the CR 2025. Half cells with LFP as the cathode, Li-metal as the anode, and Celgard 2500 as separator were assembled in the electrolytes (30 μL) with and without additive in the CR 2025. All cells were assembled in an Ar-filled universal glove box with the oxygen and water vapor pressure less than 0.1 ppm. Electrochemical impedance spectroscopy (EIS) measurements were carried out using a CHI 600E electrochemical workstation on Li||Li cells, with frequencies ranging from 0.1 to 100 kHz with an alternating voltage amplitude of 10 mV. Linear sweep voltammetry (LSV) tests were implemented at a scan rate of 1 mV s^−1^ and a voltage range from 3.0 to 5.0 V. Galvanostatic tests were measured using Neware electrochemical test system. Li||Li cells were cycled with the areal capacities of 0.5 and 3 mAh cm^−2^ at the current densities of 1 and 3 mA cm^−2^, respectively. The stability of Li||LFP full cells were cycled at 1C (170 mA g^−1^) between 2.5 and 4.0 V. Coulombic efficiency (CE) test was measured as Aurbach method [44]. Specifically, 5 mAh cm^−2^ Li was deposited on Cu foil under 1 mA cm^−2^, after which the cell was charged to 0.5 V to fully strip the active Li. Then, another 5 mAh cm^−2^ Li was deposited on the Cu surface as (${Q}_{T}$), cycling at 1 mA cm^−2^ and 0.5 mAh^−2^ ( ${Q}_{C}$) for 10 cycles, after which stripping Li to 0.5 V (Qs). CE is calculated as following Eq. (1)

1 $$\mathrm{CE}=\frac{n{Q}_{C}+Qs}{n{Q}_{C}+{Q}_{T}}$$

where *n* is the cycling number. ${Q}_{C}$, Qs, and ${Q}_{T}$ are the fixed capacity of Li, stripping capacity of Li, and capacity of Li reservoir, respectively.

The *t*_Li_^+^ was calculated by the Bruce & Vincent method, according to the following Eq. (2) [45]:

2 $$ t_{Li}^{ + } = \frac{{Iss\left( {\Delta V - I_{o} R_{0} } \right)}}{{I_{0} \left( {\Delta V - I_{ss} R_{ss} } \right)}} $$

where ${R}_{0}$ and ${R}_{ss}$ are the interfacial resistance of the Li electrode before and after the polarization, respectively. ${I}_{0}$ and ${I}_{ss}$ are the initial-state and steady-state currents during polarization, respectively. ΔV is the bias voltage. The bias voltage of chronoamperometry test of Li||Li cells was 10 mV.

### Density Functional Theory Calculation

Representative solvation configurations were derived from extensive atomistic simulations, which were adopted as starting configurations for additional density functional theory (DFT) calculation. Electrostatic potential (ESP) mapping was calculated by Multiwfn program and plotted by visual molecular dynamics (VMD) [46]. Binding energy is calculated according to the following Eq. (3):

3 $${E}_{\mathrm{binding}}={E}_{\mathrm{complex}}-({E}_{\mathrm{Li}+}+{E}_{\mathrm{solvent}})$$

where the ${E}_{\mathrm{complex}}$ is the energy of the complex formed by lithium ions and solvent molecules, ${E}_{\mathrm{Li}+}$ and ${E}_{\mathrm{solvent}}$ represent the energy of isolated lithium ions and solvent molecules, respectively.

## Results and Discussion

### Additive Effect on Performance of Li-Metal Electrodes

**Figure 1:**
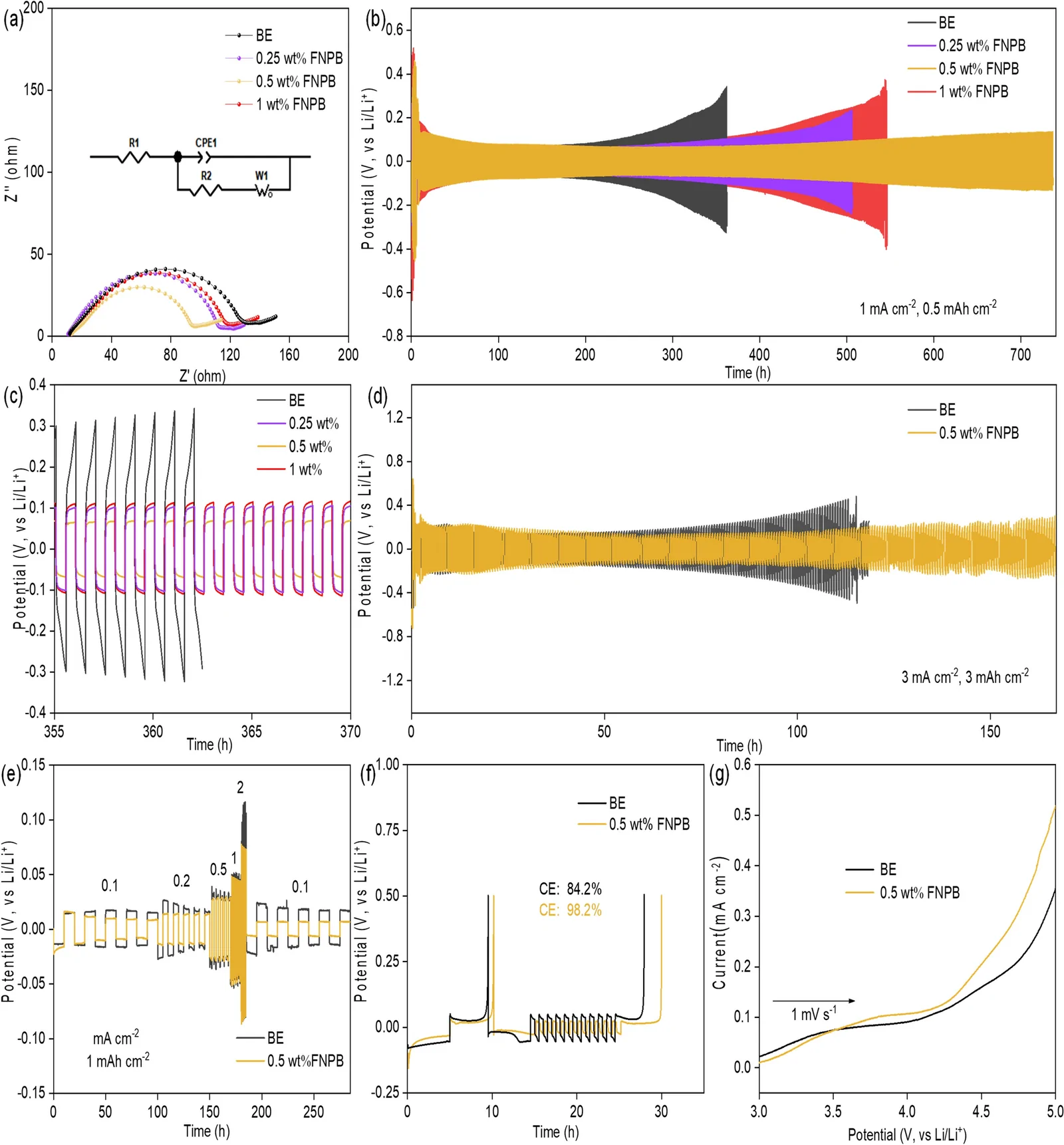
Performance of Li-metal electrodes in electrolytes with different amounts of FNPB. **a** EIS plots after 50 cycles at 1 mA cm^−2^ and 0.5 mAh cm^−2^. Plating/stripping performance at **b**, **c** 1 mA cm^−2^ and 0.5 mAh cm^−2^, **d** 3 mA cm^−2^ and 3 mAh cm^−2^, and **e** varying currents from 0.1 to 2 mA cm^−2^ and 1 mAh cm^−2^. **f** Aurbach coulombic efficiencies. **g** Anodic stability of Li-metal with the stainless-steel plate cathode revealed by LSV scanning at 1 mV s^−1^

As shown in the Nyquist plots (Fig. S1), the charge-transfer resistance (R_ct_) of Li-metal symmetric cells before cycling rises from about 150 to 450 Ohm when the amount of FNPB in the electrolyte increases from 0 to 1% in weight percentage [47] The increased R_ct_ in the EIS spectra confirm the efficiency of FNPB in altering the kinetics of electrolyte and electrolyte interphases. The similar electrolyte internal resistance (R_i_) shown by EIS spectra and the almost identical solvation effect demonstrated by similar signals of coordinated ${PF}_{6}^{-}$ at 743 cm^−1^ and solvating EC/DEC at 905 cm^−1^ in the Raman spectra (Figs. S2 and S3) indicate that the kinetics regulation of FNPB may mainly target the electrolyte interphases [48]. After initial test of 50 cycles at 1 mA cm^−2^ and 0.5 mAh cm^−2^, the decreased R_ct_ values of the Li-metal electrodes in the electrolytes with FNPB (Fig. 1a) further confirm the improved interface kinetics due to the evolution of stable SEIs [49]. Among all the electrolytes, the one with 0.5 wt% FNPB substantiates the best improved interface kinetics with R_ct_ of 95 Ohm, in sharp contrast to 133 Ohm of BE.

Except for the kinetics process, the plating/stripping stability of Li-metal electrodes is also enhanced after FNPB addition. When galvanostatic plating/stripping at 1 mA cm^−2^ and 0.5 mAh cm^−2^ (Fig. 1b, c), the Li-metal electrodes in BE stably persist for only 200 h, and exhibit ever-increasing overpotentials. The severe increased polarization overpotential up to 0.3 V at 350 h leads to the short circuit of the Li-metal symmetric cell, demonstrating ineffective lithium dendrite suppression. In contrast, the cycling stability of the Li-metal electrodes in FNPB is generally enhanced, showing lowered overpotentials of 0.11, 0.063, and 0.105 V at 350 h for electrolytes with 0.25, 0.5, and 1 wt% FNPB, respectively. The reduced polarization of lithium plating/stripping process attributes to the enhanced kinetics of SEIs forming in the initial cycles, as indicated by the initial large overpotentials due to the decomposition of the FNPB additive. The calculated binding energies of Li-metal with FNPB, EC, and DEC (Fig. S4) show the priority of reaction between Li and FNPB. The molecular electrostatic potential surface simulation (Fig. S5) further demonstrates the possible reaction sites of -NO_2_, -BO_2_-, and -F, with gradually decreasing negative potentials, readily producing multiple F-rich/N-rich interphases (Fig. S6) with enhanced stability and diffusion kinetics. Notably, Li-metal electrodes in electrolytes with 0.5 wt% FNPB maintain > 700 h stable cycling performance at 1 mA cm^−2^ and 0.5 mAh cm^−2^ without short circuiting, indicating the effectively suppressing of dendrite growth. Moreover, the lower polarization overpotentials and enhanced cycling stability at 3 mA cm^−2^ and 3 mAh cm^−2^, and at varying rates of 0.1–2 mA cm^−2^ and 1 mAh cm^−2^ (Fig. 1d, e) furthers substantiate the effectiveness of FNPB in evolving SEIs with rapid Li^+^ transfer and enhance interfacial stability at different cycling conditions, as verified in the significantly elevated Li^+^ transference number from 0.17 to 0.89 (Figs. S7 and S8).

The utilization efficiency of Li is determined by CE of Li||Cu asymmetric cells according to the Aurbach method [44]. As shown in Fig. 1f, the CE of the cell with BE is 84.2%, while that with 0.5 wt% FNPB is elevated to 98.2%, implying that the addition of FNPB probably avails to the evolution of stable SEIs and inhibits continuous side reactions between Li-metal and the electrolyte. The LSV (Fig. 1g) is performed to explore the anodic stability of Li-metal electrodes in different electrolytes. The cell with BE exhibits an obvious increase in polarization current at 4.3 V (vs. Li/Li^+^) due to the oxidative decomposition of carbonate components at the stainless-steel plate cathode. The onset potential shifts to about 3.75 V (vs. Li/Li^+^) for the cell with 0.5 wt% FNPB, indicating that FNPB decomposes preferentially and probably evolves into protective CEIs on the surface of the cathode, consistent with the theoretical calculation results of binding energy mentioned above [50–52]. Figures S9 and S10 displayed that the contact angle decreases from 36.66° of BE to 34.93° of 0.5 wt% FNPB, indicating enhanced wetting ability of the electrolyte after FNPB addition. The good wettability helps to achieve consistent distribution of lithium-ion fluxes favorable for fast kinetics and uniform Li deposition.

### Additive Effect on Morphologies of Li Deposit

**Figure 2:**
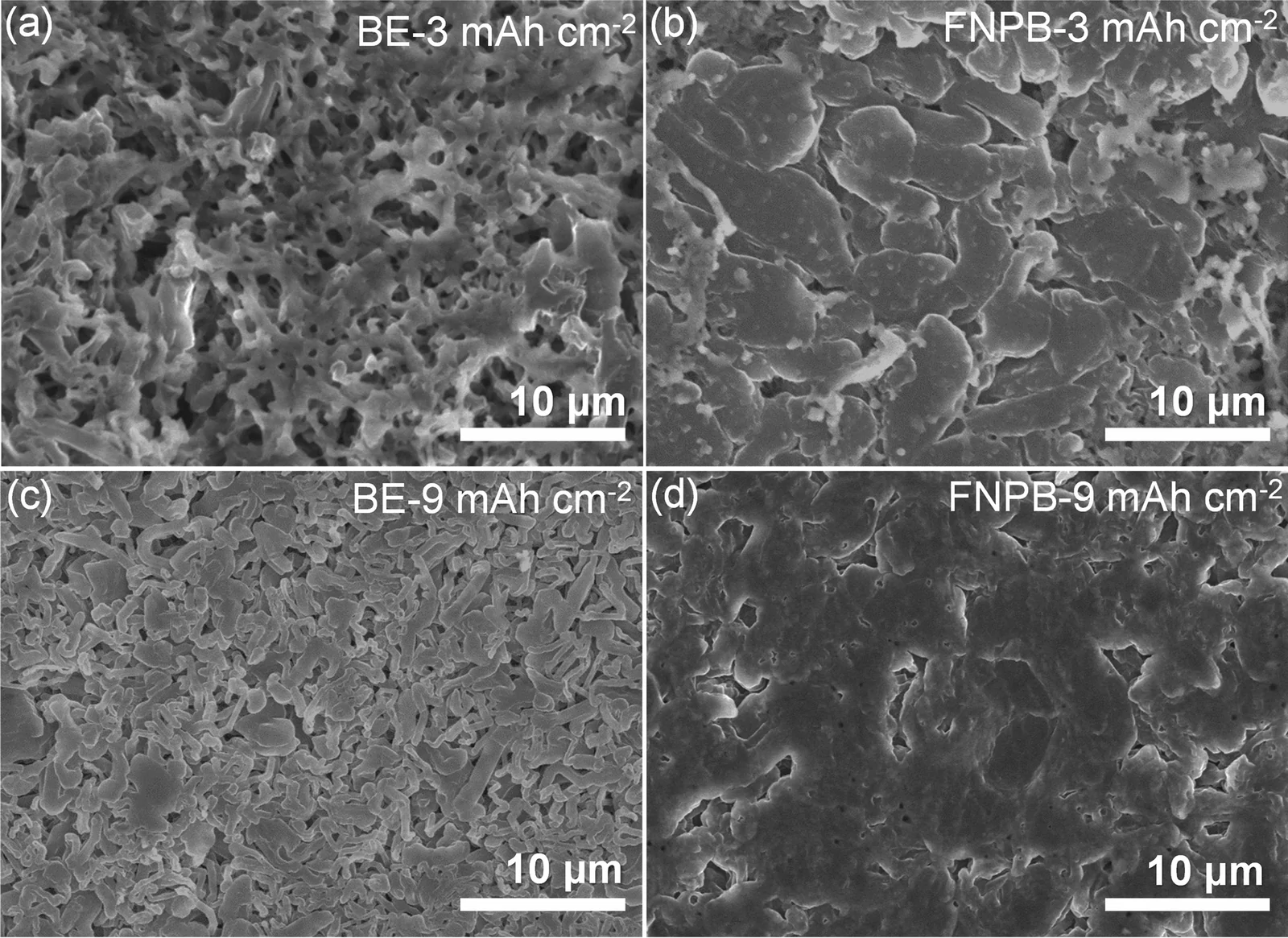
SEM images of Li deposit on Cu substrates after plating in different electrolytes at 1 mA cm^−2^ for **a**, **b** 3 h, **c**, **d** 9 h

The morphology and microstructure of Li deposit present important insights into the reversibility of Li-metal electrodes. The SEM images of the deposit on the Cu substrates at 1 mA cm^−2^ (Fig. 2) show that Li deposit plated in BE for 3h, i.e., 3 mAh cm^−2^, appears as highly porous needle-like products ranging from nanometers to micrometers (Fig. 2a), known as Li dendrites. Although the deposition layer densifies as the dendrites aggregate after plating from 3 to 9 h (Fig. 2c), the dendritic growth behavior maintains due to the lack of an effective electrolyte interphase layer, easily piercing separators and causing cell short circuit. In contrast, a quite different two-dimension (2D) planar growth behavior is observed for the Li deposit plated in FNPB (Fig. 2b), which leads to significantly elevated densification and alleviates volume effect as the plating process continues (Fig. 2d). The chronoamperometry analysis (Fig. S11) also reveals that the initial nucleation current has been significantly increased after FNPB addition, indicating a greater number of nucleation sites and more uniform lithium deposition. The compact deposition layer also avails to reduced evolution of “dead Li” due to lose-of-connect, quite different from easy disconnection of partial Li in the porous dendrites, consistent to the elevated Li utilization efficiency for Li plating/stripping in FNPB. The results indicate that the FNPB additive effectively regulates the growth behavior of Li-metal, promoting the formation of densification Li deposit, which contributes to enhanced cycling stability of Li-metal electrodes.

**Figure 3:**
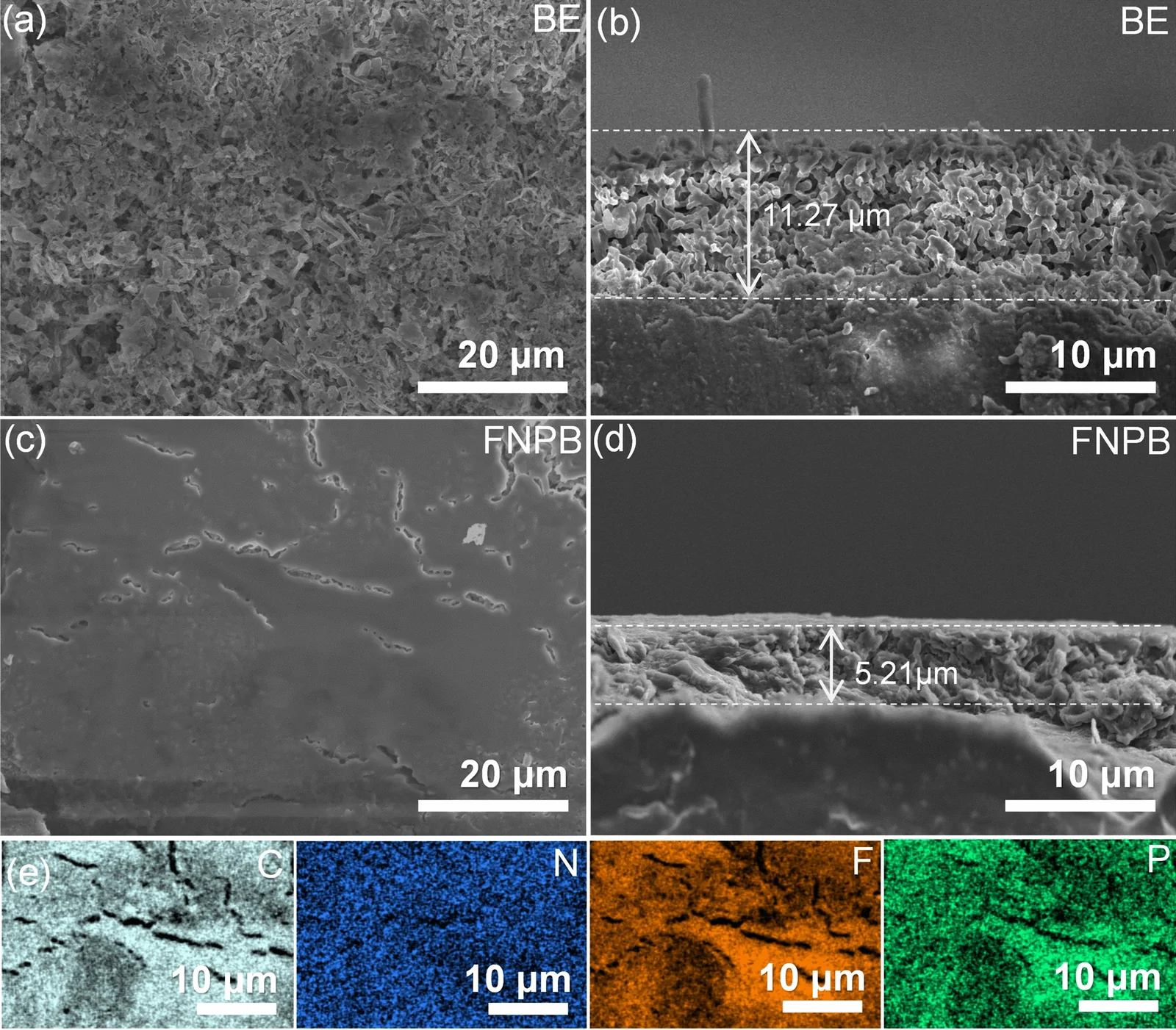
**a**, **c** Top-view, **b**, **d** cross-sectional SEM images of Li electrodes after plating/stripping at 1 mA cm^−2^ for 50 cycles in different electrolytes, and **e** the corresponding EDS element mapping images

The SEM images after 50 plating/stripping cycles (Fig. 3) are also presented to further explore the different deposition behaviors. After repeatedly plating/stripping processes, the top-view SEM image (Fig. 3a) displays that the deposit layer of Li-metal electrodes in BE still hold the dendrite-like morphology different from the pristine one, showing abundant pores at micrometer scale (Figs. S12 and S13). No obvious electrolyte interphase layer with a continuous or consistent structure can be observed, implying uneven SEIs or uncontrolled electrolyte decomposition during the plating/striping process. The corresponding cross-sectional SEM image in Fig. 3b further confirms a large thickness of 11.27 μm for the deposit layer, verifying inconsistent deposition of Li with very poor compactness in the dendrite growth. While the top-view SEM image of the Li-metal electrode in FNPB (Fig. 3c) exhibits a uniform and smooth appearance. Moreover, the cross-sectional SEM image (Fig. 3d) shows a significantly lowered thickness of only 5.21 μm. The uniform electrolyte interphase layer and the very compact deposit together verify that FNPB is an effective additive to induce the formation of a uniform SEI layer, which regulates dense and uniform Li deposition in planar growth manner, inhibiting dendrite growth, side reactions, and ultimately leading to higher CE and longer cycling stability. The corresponding SEM EDS element mapping images (Fig. 3e) display that the SEI layer is characteristic of compactly packed monodisperse particles (Fig. S14) with uniformly distributed C, N, F, and P elements, attributed to uniform distribution of FNPB-derivatives and their interaction with the electrolyte.

### Additive Effect on the Electrolyte Interphases of Li-Metal Electrodes

**Figure 4:**
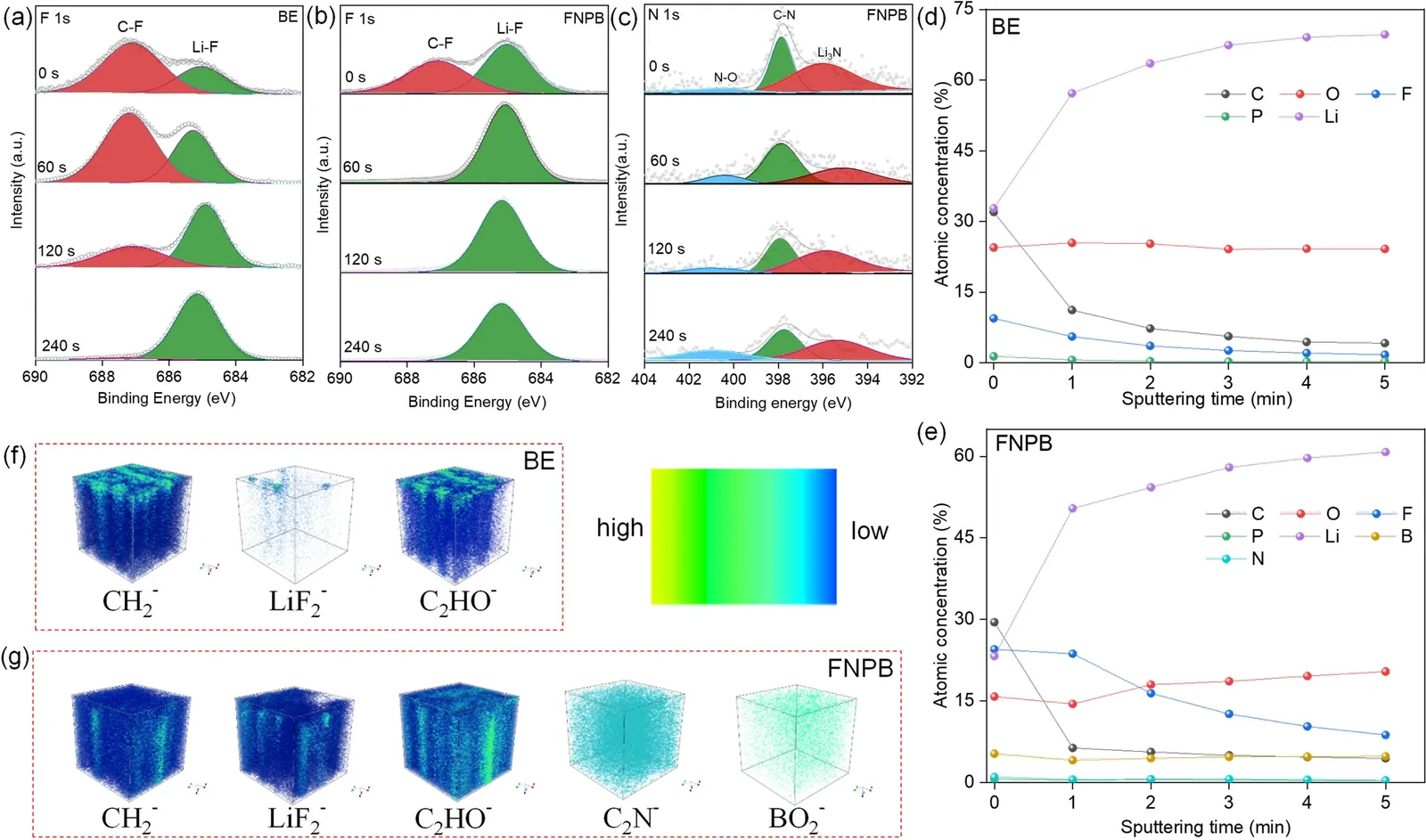
Interphases of Li-metal electrodes after 50 cycles in different electrolytes. Deconvoluted **a**, **b** F 1*s*, and **c** N 1*s* spectra of XPS depth profiling, and **d**, **e** the corresponding quantitative elemental analysis profiles. **f**, **g** 3D render overlay images for various fragments of TOF–SIMS depth profiling

The interphase species in the SEIs are further analyzed by XPS and TOF–SIMS. The Li-metal electrodes cycled in electrolytes with/without the FNPB additive show similar SEIs with both the organic and inorganic components. The difference lies in the types, distribution, and content of the components. Specifically, the F 1*s* XPS depth profiles (Fig. 4a, b) after different durations of Ar^+^ plasma etching display similar deconvoluted peaks at 684.8 eV from Li-F bonding of inorganic LiF, and 686.8 eV from C-F bonding from organic species [53]. The variation of their normalized intensities exhibits that the intensities of C-F signal progressively decrease for both SEIs, but the one obtained in FNPB shows negligible C–F intensity after 60 s sputtering, implying the formation of LiF-rich interphase layer. Concurrently, the deconvoluted N 1*s* XPS spectra in Figs. 4c and S15 displayed N–O (~ 400.8 eV), C–N (~ 397.8 eV), and Li–N (~ 395.2 eV) bonding signals exclusively for the SEI in FNPB system, confirming the involvement of nitrogen-containing groups (–NO_2_) from FNPB in evolving into inorganic Li_3_N and organic C–N/N–O species [54]. The emergence of N species in the SEI, e.g., Li_3_N as an excellent ionic conductor, compensates for the low ionic conductivity of LiF, guaranteeing the good ion conductivity of the SEI layer for fast-charging process.

The LiF-rich interphase in SEIs for FNPB is also confirmed by the remarkable intensities of LiF signals from LiF at 52.5 eV in the deconvoluted Li 1*s* XPS spectra (Figs. S16 and S17), much stronger than the counterparts for BE throughout the plasma sputtering. Due to its excellent electronic insulation performance and high interfacial energy, LiF is regarded as a key SEI component for inhibiting Li dendrite growth and ensuring high CE [55]. Besides, the deconvoluted B 1*s* XPS spectrum (Fig. S18) also indicates the evolution of borate or fluoborite interphases, e.g., LiB_x_O_y_, with relevant signals of B-O bonding at 191.7 eV and B-F bonding at 194.3 eV, respectively. These inorganic interphases are also beneficial components, inhibiting the consumption of the bulk electrolyte and the growth of lithium dendrites [56]. In the XPS quantitative analysis (Fig. 4d, e), the faster decrease in the content of C species (mainly from organic interphase) for SEIs in FNPB than those in BE, and the slower decrease in the content of F and B species (mainly from inorganic interphase) for SEIs in FNPB than those in BE as the sputtering lasts, also substantiate the inorganic-rich interphases for the SEI evolved in FNPB.

The spatial distribution of the interphase species is further revealed by the corresponding three-dimensional (3D) render overlay images of TOF–SIMS depth profiling (Fig. 4f, g). The extremely uneven distribution of the strong signals of CH_2_^−^ and C_2_HO^−^ fragments from organic species, and the very weak signal of LiF_2_^−^ fragment from LiF indicates an organic-rich SEI in the Li-metal electrodes cycled in BE. In contrast, relatively similar intensities of the distribution of CH_2_^−^, C_2_HO^−^, and LiF_2_^−^ signals substantiate a uniform SEI with relatively high content of inorganic LiF. Moreover, the emerging C_2_N^−^ fragment from C–N organic species, and LiNO_2_^−^ and BO_2_^−^ fragments from inorganic nitride and borate species (Figs. 4g and S19) indicate that the FNPB additive also introduces abundant N-/B-based interphases, demonstrating the effectiveness of –NO_2_/–B(OH)_2_/–F groups in forming inorganic-rich SEIs. The SEI layer with uniform distribution of organic and abundant inorganic species accounts for the suppressed lithium dendrite growth.

### Additive Effect on the Performance of LMBs

**Figure 5:**
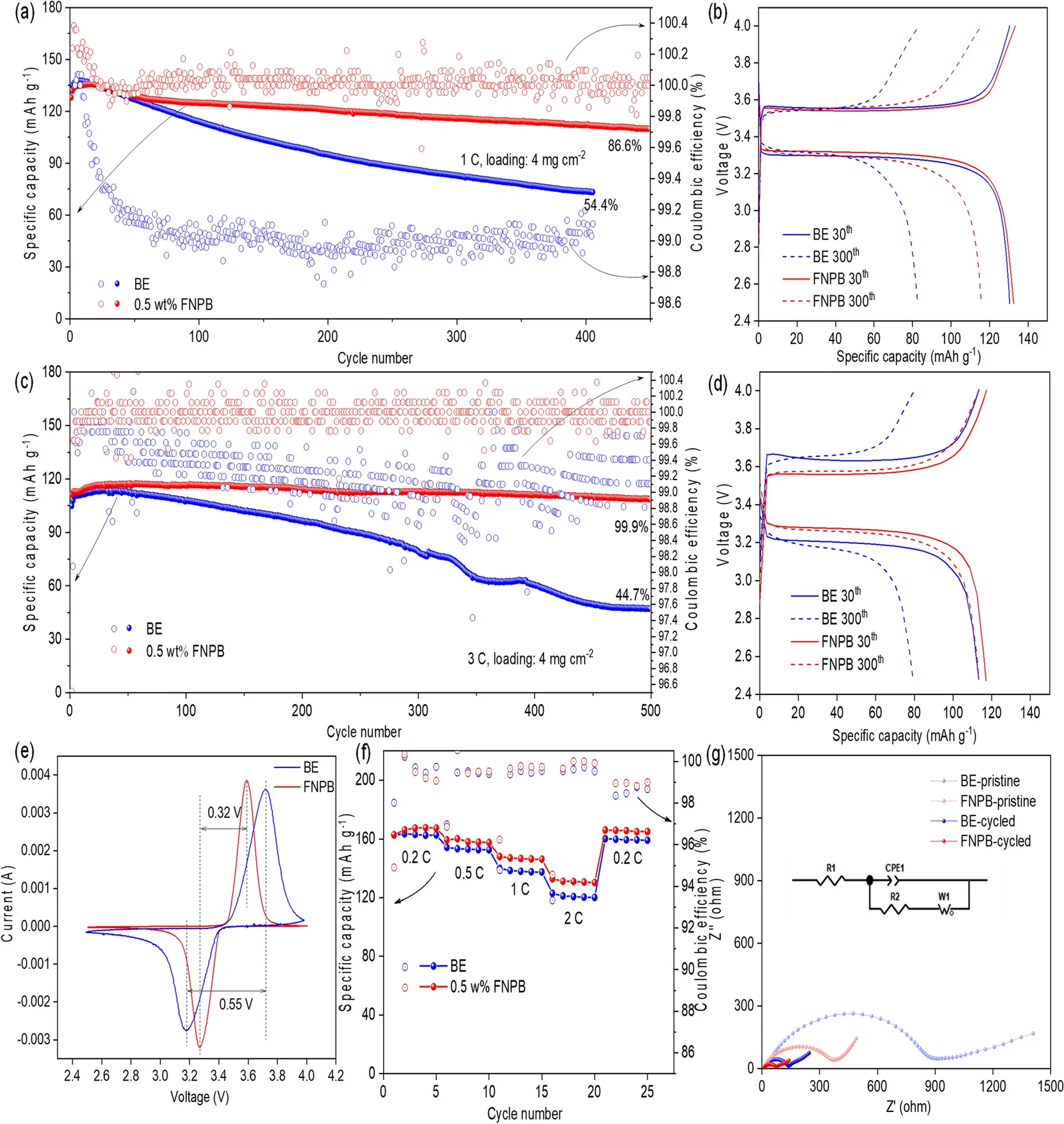
Electrochemical performance of LMBs with the LFP cathodes and different electrolytes cycling at 2.5–4.0 V. **a**, **c** Cycling performance and **b, d** voltage profiles tested at different C rates. **e** CV curves at 0.1 mV s^−1^, **f** rate performance, and **g** EIS plots

Benefiting from the N-/F-rich SEI inducing compact and uniform Li deposition in FNPB, the LMBs with the commercial LFP cathodes demonstrate enhanced electrochemical performance. Specifically, at a window voltage of 2.5–4.0 V, both LMBs deliver similar initial reversible capacity of about 130 mAh g^−1^ at 1C (1C = 170 mA g^−1^, Fig. 5a, b) due to similarly low deposition overpotentials of the Li-metal anodes at the initial cycles. However, comparing to the LMB with BE, the one with NFPB exhibits enhanced cycling stability, demonstrating 86.6% capacity retention after 450 cycles at 1C, far superior to only 54.4% after 400 cycles of the former. The remarkable advantage of fast-charging performance is even prominent at 3C (Fig. 5c, d) rate. The initial coulombic efficiency increases from 76.1% to 96.5% after 0.5 wt% FNPB addition, substantiating the efficiency of the additive in inhibiting uncontrolled electrolyte decomposition [35]. Simultaneously, a reversible capacity of 111 mAh g^−1^ is achieved in the LMB with FNPB, which maintains 99.9% capacity retention after 500 cycles, far superior to only 44.7% of the control one with BE. Notably, the LMBs with FNPB also maintained > 99.9% coulombic efficiencies throughout the cycling processes at both rates, while the coulombic efficiencies only hold at 99.0% for the control ones with BE due to continuous electrolyte decomposition or loss of active species due to ineffective electrolyte interfaces.

The remarkably alleviated voltage hysteresis in the charge/discharge plateaus and cyclic voltammetry curves (Fig. 5d, e) further indicate that the kinetics process is significantly improved. Except for cycling stability and power performance, the ion storage capability at relatively small rates has also been strengthened due to high Li utilization efficiency endowed by the effective electrolyte interphases. The LMBs with FNPB (Fig. 5f) deliver reversible discharge specific capacities of 167, 158, 147, and 131 mAh g^−1^ at 0.2C, 0.5C, 1C, and 2C, respectively, generally higher than those of the control ones. The enhanced fast kinetics in FNPB is highly relevant to the significantly reduced charge-transfer resistance from 373 to 74 Ohm (vs. 895 to 135 Ohm of the control ones) of the electrolyte interphase layers (Fig. 5g). Overall, the FNPB-induced fast-kinetics N-/F-rich interphase layers remarkably improve the cycling stability and ion storage capability of LMBs under fast-charge conditions.

### Additive Effect on the Electrolyte Interphases of LFP Cathodes

**Figure 6:**
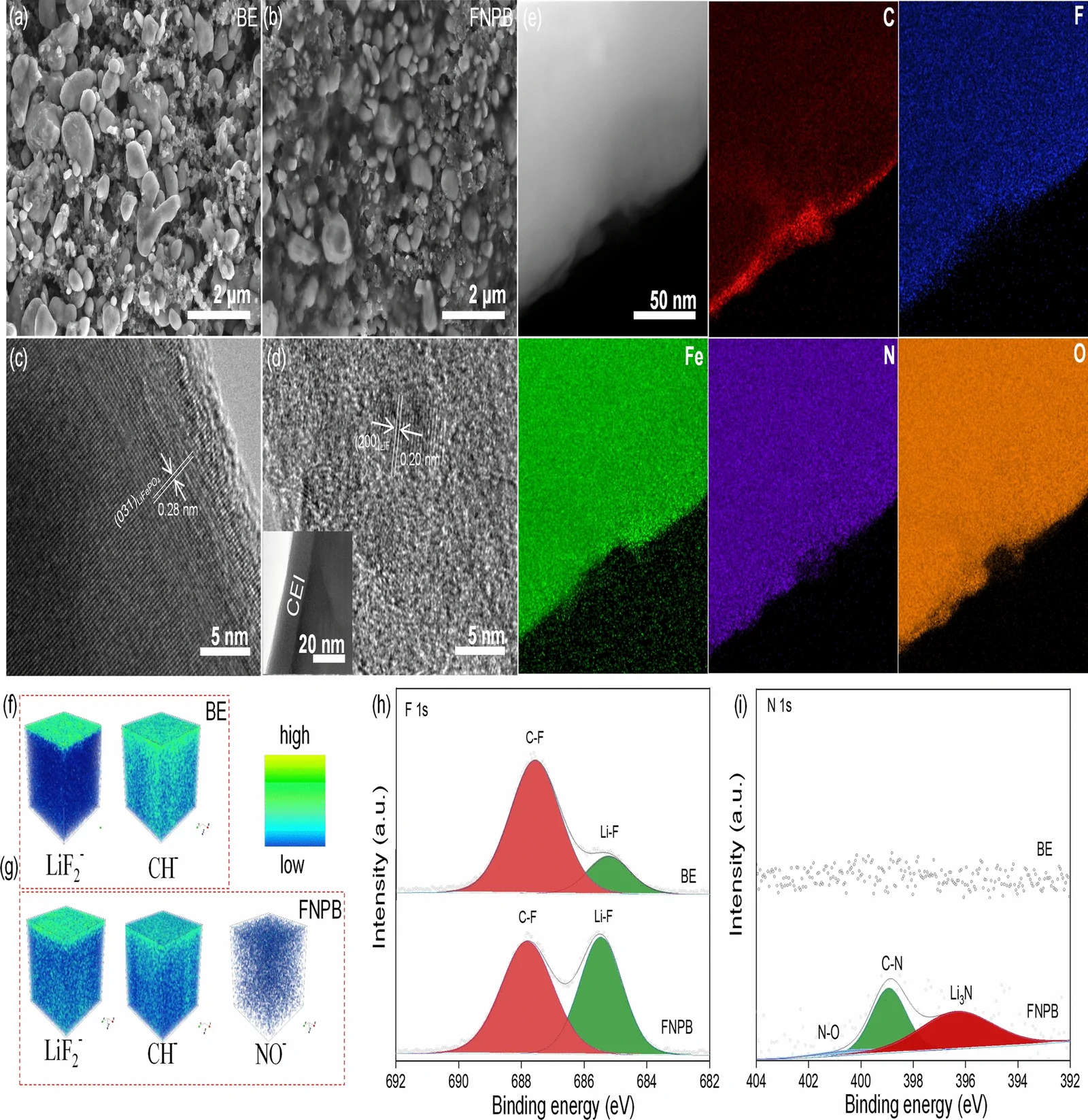
**a**, **b** SEM, **c**, **d** TEM and HRTEM, **e** HAADF-STEM and EDS element mapping images of LFP cathodes cycled in different electrolytes after 100 cycles. TOF–SIMS 3D render overlay images of LFP cathodes cycled in **f** BE and **g** FNPB after 50 cycles. **h**, **i** F 1*s* and N 1*s* XPS spectra of LFP cathodes cycled after 50 cycles

The LFP cathodes after 100 cycles are also characterized to further get insights into the performance improvement. The SEM images (Fig. 6a, b) exhibit similar irregular particles of commercial LFP at micrometer scale. Differently, the particles cycled in FNPB show relatively uniform gel-like film encapsulation, indicating the formation of uniform CEI layer. The corresponding TEM and high-resolution TEM (HRTEM) images (Fig. 6c, d) further confirm the film of uniform thickness of 15–20 nm, featuring abundant nanocrystals of about 5 nm distributed in the amorphous species. The nanocrystals exhibit well-defined lattice fringes with an interplanar spacing of 0.2 nm, ascribing to (200) planes of cubic LiF. In contrast, no consistent covering can be observed outer the particles of LFP cycled in BE. The uniform CEI layer is also confirmed in the high angle annual dark-field scanning TEM (HAADF-STEM) image (Fig. 6e), showing clear encapsulation layer outer the LFP particle with less pronounced contrast. The corresponding EDS element mapping images display remarkable aggregation of C, O, and F elements in the CEI layer, probably from amorphous organic carbon and crystalline LiF.

TOF–SIMS 3D render overlay images (Fig. 6f, g) for depth profiling reveal that both cathodes show similar signal intensity of LiF_2_^−^ fragments from LiF, indicating the formation of LiF interphase due to initial electrolyte decomposition. Differently, comparing to the cathode cycled in BE, the one in FNPB shows slower decrease in LiF content from outer to inner layer, verifying much more and compacter LiF interphases. Simultaneously, the change of CH^−^ fragment from organic carbon implies the latter features fewer organic species. The emergence of NO^−^ fragment indicates the existence of nitrite interphases in the CEI layer. The F-/N-rich interphases in the CEI are also corroborated by the local deconvoluted F 1*s* and N 1*s* XPS spectra (Fig. 6h, i), showing intensified LiF bonding signal at about 685 eV, and N-Li (396 eV), C-N (399 eV), N–O (401 eV) bonding signals from Li_3_N, LiNO_2_ and organic C-N species [57]. The stable dense CEI formed by introducing FNPB can thus provide enduring protection for the cathode, maintain the integrity of LFP. Obviously, the FNPB additive facilitates the evolution N-/F-rich inorganic interphases in both the anode and the cathode of LMBs, contributing to uniform SEI and CEI layers simultaneously, which not only guarantee suppressed lithium dendrite growth, but also alleviated uncontrolled electrolyte decomposition and loss of active species.

## Conclusion

In summary, FNPB has been demonstrated as an electrolyte additive to significantly enhance the performance of LMBs with conventional EC/DEC electrolytes. During the initial cycles, small quantities of additives decompose sacrificially, forming electrochemically stable SEIs and CEIs. The uniform N-/F-rich interphase layers effectively prevent excessive electrolyte decomposition, inhibit dendrite formation during cycling and thereby enhance cycling stability and coulombic efficiency of LMBs. The work implies that proper electrolyte additive matters very much for LMBs at fast-charging processes.

## Supplementary Information

Below is the link to the electronic supplementary material.

Supplementary file1 (DOCX 1218 KB)
