# Supplementary material for: Uniform Fast-Kinetic Anode/Cathode Electrolyte Interphases Enable High Performance 3C Li-Metal Batteries with > 99.9% Coulombic Efficiencies
Source: Nanomicro Lett. 2026 Feb 9;18:243. doi: 10.1007/s40820-026-02088-w (PMC12886608; doi:10.1007/s40820-026-02088-w)
Supplement: Supplementary file 1 — Supplementary file1 (DOCX 1218 KB) [file 40820_2026_2088_MOESM1_ESM.docx]

Supporting Information for

**Uniform Fast-Kinetic Anode/Cathode Electrolyte Interphases Enable High Performance 3C Li-Metal Batteries with >99.9% Coulombic Efficiencies**

Qingyang Cao^1^, Danchen Fu^1^, Xuedong He^1^, Yaohua Huang^1^, Ningning Yao^1^, Chunyu Song^1^, Huawei Song^1^* and Chengxin Wang^1^*

^1^State Key Laboratory of Optoelectronic Materials and Technologies, School of Materials Science and Engineering, Sun Yat-Sen (Zhongshan) University, Guangzhou 510275, People’s Republic of China

*Corresponding authors. E-mail: [songhw5@mail.sysu.edu.cn](mailto:songhw5@mail.sysu.edu.cn) (Huawei Song); [wchengx@mail.sysu.edu.cn](mailto:wchengx@mail.sysu.edu.cn) (Chengxin Wang)

**Supplementary Figures and Tables**


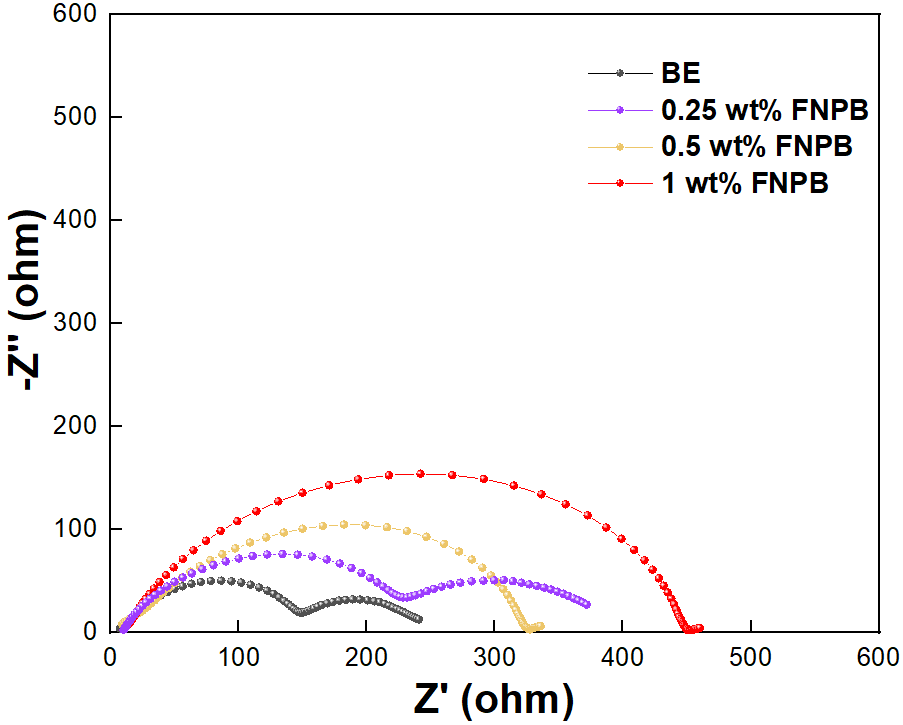


**Fig. S1** EIS plots of pristine Li-metal symmetric cells with electrolytes containing different amounts of FNPB


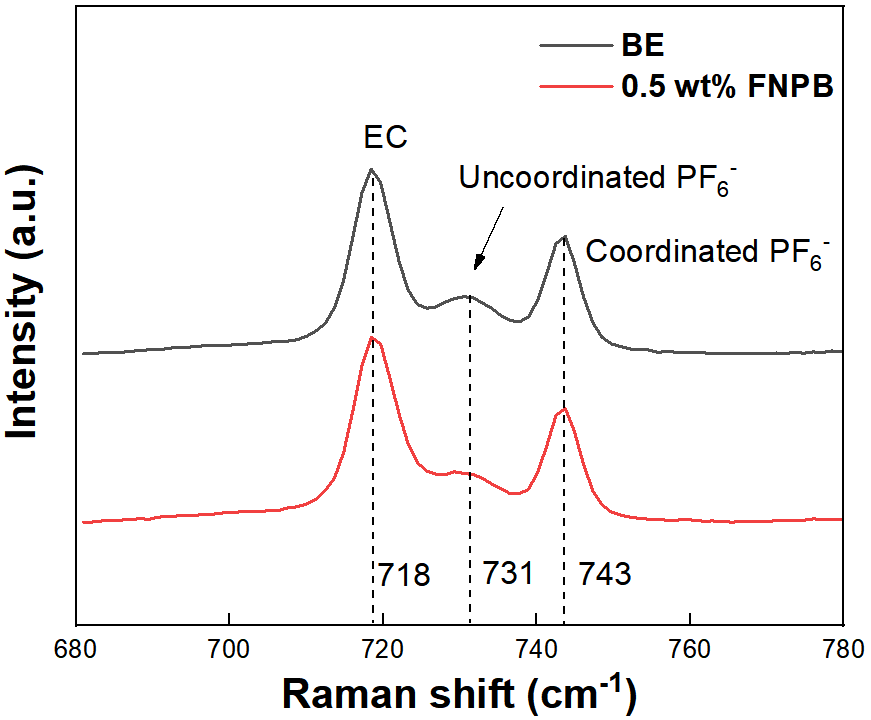


**Fig. S2** Local anion signals in Raman spectra of electrolyte with/without FNPB


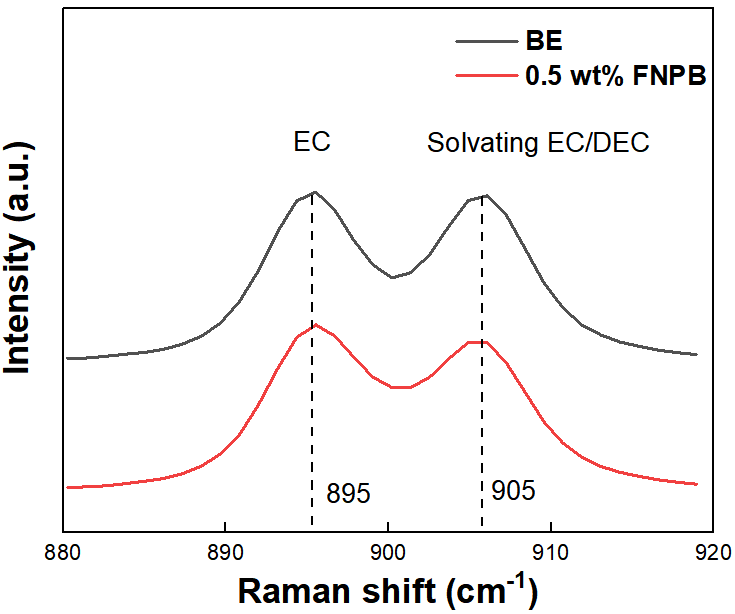


**Fig. S3** Local cation signals in Raman spectra of electrolyte with/without FNPB

**Fig. S4** The Binding Energies of Li metal with FNPB, EC and DEC


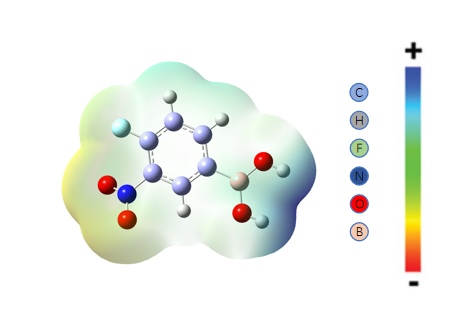


**Fig. S5** Electrostatic potential surface, with yellow and blue areas representing negative and positive charges, respectively


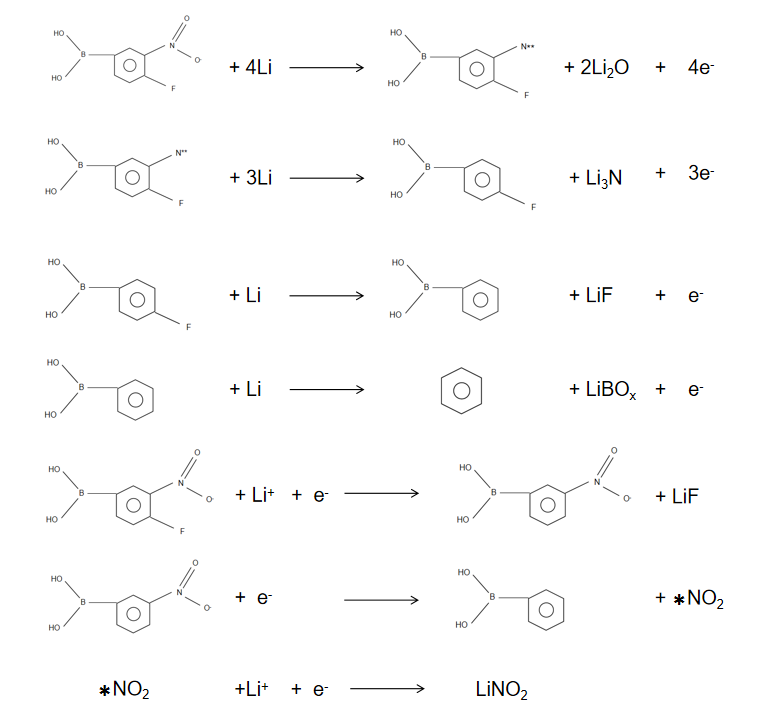


**Fig. S6** One possible reaction pathway between FNPB and Li metal and LiFePO_4_


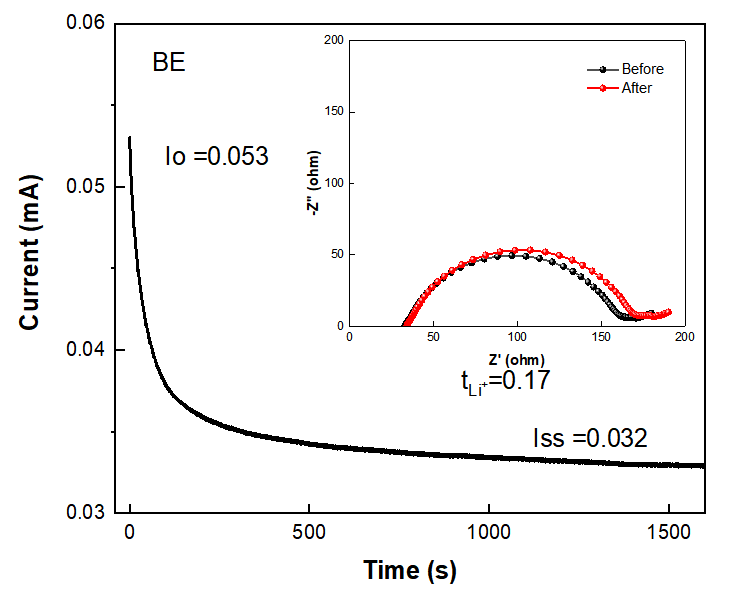


**Fig. S7** Current-time plots of Li-metal symmetric cells with BE after polarization at a constant potential of 10 mV for 1600 s. The inset plots are the impedance spectra before and after the polarization


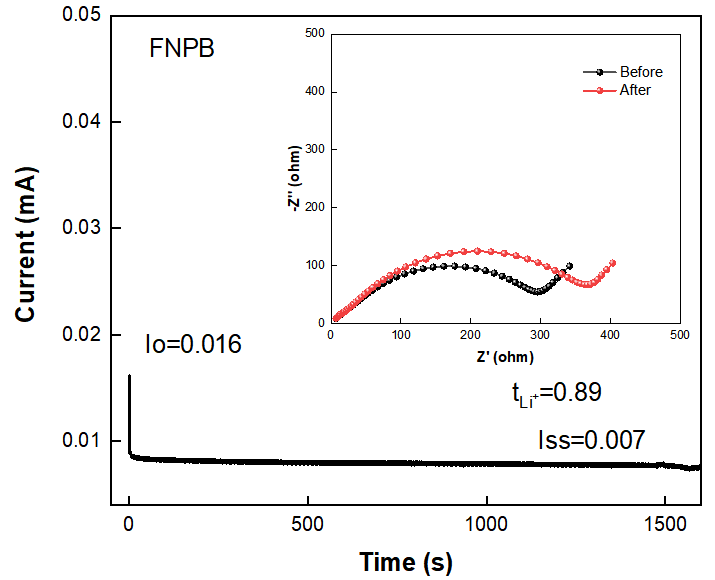


**Fig. S8** Current-time plots of Li-metal symmetric cells with electrolyte containing FNPB additive after polarization at a constant potential of 10 mV for 1600 s. The inset plots are the impedance spectra before and after the polarization


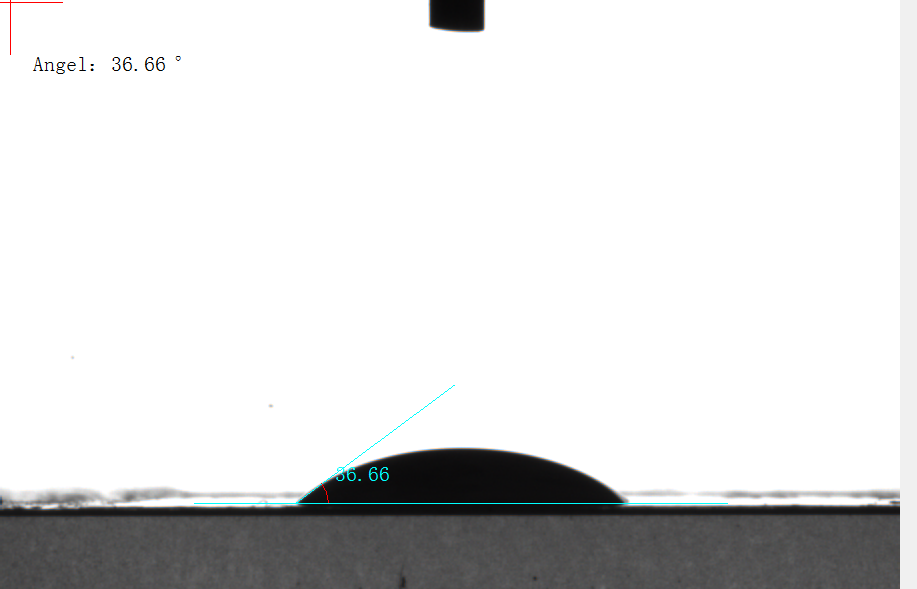


**Fig. S9** Contact angle measurement of BE


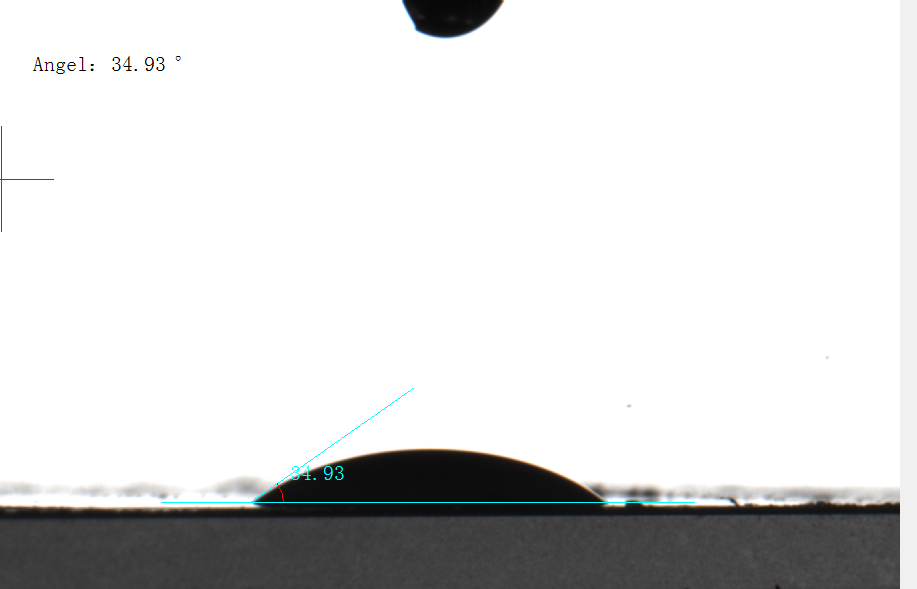


**Fig. S10** Contact angle measurement of electrolyte after adding FNPB

**Fig. S11** Chronoamperoetry test of Li||Li cells in BE and FNPB electrolyte
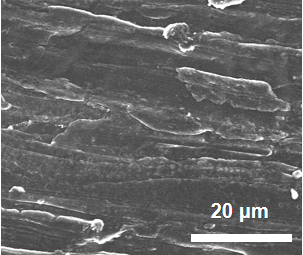


**Fig. S12** SEM image of the pristine Li-metal electrode


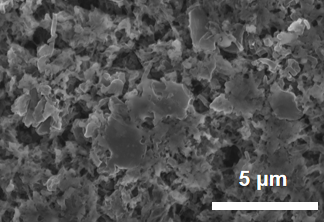


**Fig. S13** SEM images of Li-metal electrodes plating/stripping in BE at 1 mA cm^-2^ for 50 cycles


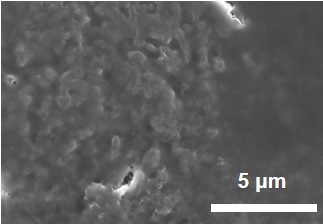


**Fig. S14** SEM images of Li-metal electrodes plating/stripping in FNPB at 1 mA cm^-2^ for 50 cycles


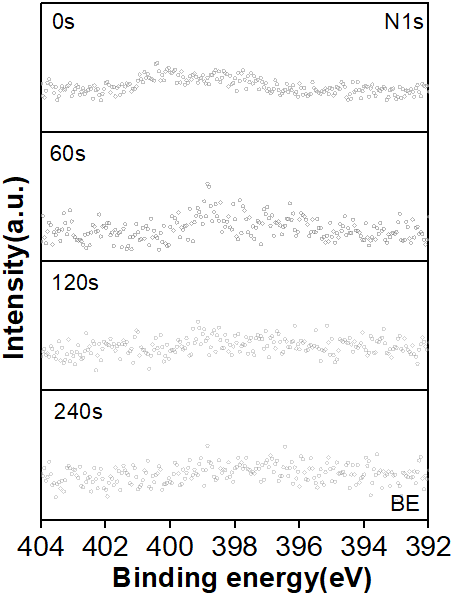


**Fig. S15** N 1s XPS depth profiles of the Li-metal electrode cycled in BE after 50 cycles

**Fig. S16** Li 1s XPS depth profiles of the Li-metal electrode cycled in BE after 50 cycles

**Fig. S17** Li 1s XPS depth profiles of the Li-metal electrode cycled in FNPB after 50 cycles


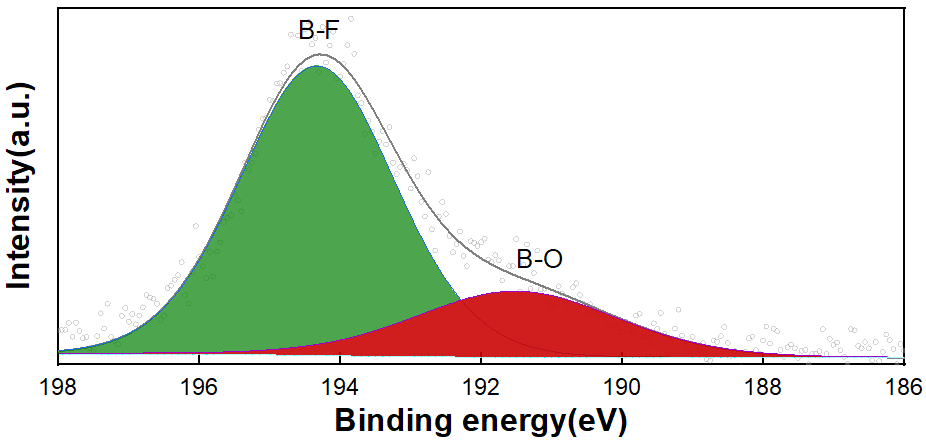
**Fig. S18** B 1s XPS spectrum of the Li-metal electrode cycled in FNPB after 50 cycles


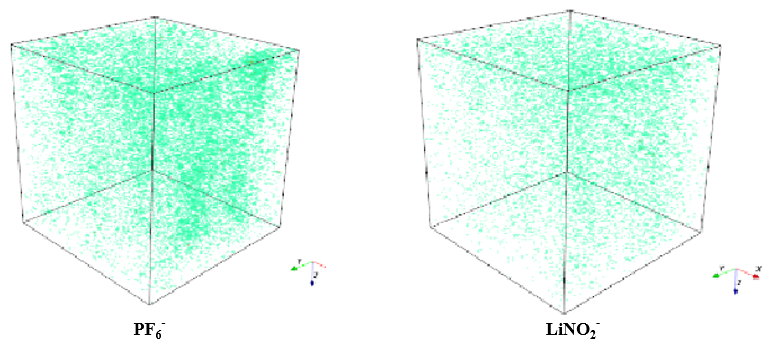


**Fig. S19** TOF-SIMS 3D render overlay images of LiNO_2_^-^ signals for the Li metal electrode cycled in FNPB after 50 cycles

**Table S1** Performance comparison of Li-metal batteries with different electrolyte additives

| Electrolyte composition | Positive electrode | Voltages  (V) | Performance | CE | References |
| --- | --- | --- | --- | --- | --- |
| 1 M LiPF_6_ in  EC/DEC (TDFA),100 μL | LFP (3 mg cm^-^) | 2.5-4.2 | 300 cycle (89.4%), 1C | / | [S1] |
| 2M LiFSI in DOL with 3vol% TEB | LFP (2.64 mg cm^-2^) | 2.5-3.8 | 500 cycle (80.0%), C/2 | 0.5mA  98.49% | [S2] |
| 1 M LiTFSI+0.04 M CH_3_OK  +2%LiNO_3_ in DME/DOL | LFP (10.5 mg cm^-2^) | 2.5-3.8 | 350 cycle (91.5%), C/2 | / | [S3] |
| 1 M LiTFSI/DOL-DME (1:1 vol%) with 1 wt% LiNO_3_(4% 2,2,2-TFTF) | LFP (1.8 mg cm^-2^) | 2.4-4.2 | 500 cycle (86.0%), | / | [S4] |
| 1 M LiPF_6_ in EC/DMC (1:1 vol%) with 1% LiNO_3_ +0.5% HFT | LFP (3 mg cm^-2^) | 2.5-4.2 | 500cycle (<80.0%),2C | 1mA  97.1% | [S5] |
| 0.6M LiFSI+0.4M LiBOB  in EC/EMC(3:7)(B-FC) | NMC622(1.4 mAh cm^-2^) | 3.0-4.5 | 300 cycle (73.1%), C/2 | / | [S6] |
| 1 M LiPF_6_ in DEC : EC : EMC (2:3:5 vol%) with 1wt% LiDFP +0.3wt% LiNO_3_ | LFP (1.25 mAh cm^-2^) | 2.2-4.1 | 350 cycle (88.0%),1C | 1mA  98.2% | [S7] |
| 1 M LiPF_6_ in EC/DEC (FNPB),30 μL | LFP (4 mg cm^-2^) | 2.5-4.0 | 500 cycle (99.9%), 3C | 1mA  98.2% | This work |

Supplementary References

1. E. Park, Y.-H. Lee, S.-H. Huh, J. Huh, Y.-E. Sung et al., Bifunctional trimethylsilyl-modified fluorinated ester additive for LiF-rich solid electrolyte interphase in lithium metal batteries. Energy Storage Mater. **78**, 104271 (2025). <https://doi.org/10.1016/j.ensm.2025.104271>
2. Y. Guo, Y. Huang, H. Hu, Y. Chen, X. Cai et al., Anion-anchoring enabling fast Li+ transport within wide temperature. Nano Energy **147**, 111568 (2026). <https://doi.org/10.1016/j.nanoen.2025.111568>
3. J. Yang, M. Li, Z. Sun, X. Lian, Y. Wang et al., Prolonging the cycling lifetime of lithium metal batteries with a monolithic and inorganic-rich solid electrolyte interphase. Energy Environ. Sci. **16**(9), 3837–3846 (2023). <https://doi.org/10.1039/d3ee00161j>
4. S. Yang, M. Hao, Z. Wang, Z. Xie, Z. Cai et al., 2, 2, 2-Trifluoroethyl trifluoroacetate as effective electrolyte additive for uniform Li deposition in lithium metal batteries. Chem. Eng. J. **435**, 134897 (2022). <https://doi.org/10.1016/j.cej.2022.134897>
5. J. Jiang, M. Li, X. Liu, J. Yi, Y. Jiang et al., Multifunctional additives to realize dendrite-free lithium deposition in carbonate electrolytes toward low-temperature Li metal batteries. Adv. Energy Mater. **14**(27), 2400365 (2024). <https://doi.org/10.1002/aenm.202400365>
6. J. Han, C. Park, D. Jin, S. Kim, C.B. Dzakpasu et al., A bis(2-fluoroethyl) carbonate as a new electrolyte additive for enhancing the long-term cycle performance of Li-metal batteries. J. Electrochem. Soc. **170**(2), 020529 (2023). <https://doi.org/10.1149/1945-7111/acbca1>
7. M. Li, C. Chen, H. Luo, Q. Xu, K. Yan et al., Constructing an inorganic-rich solid electrolyte interphase by adjusting electrolyte additives for stable Li metal anodes. J. Mater. Chem. A **12**(17), 10072–10080 (2024). <https://doi.org/10.1039/d3ta07655e>
